# Junk Food on Demand: A Cross-Sectional Analysis of the Nutritional Quality of Popular Online Food Delivery Outlets in Australia and New Zealand

**DOI:** 10.3390/nu12103107

**Published:** 2020-10-12

**Authors:** Stephanie R. Partridge, Alice A. Gibson, Rajshri Roy, Jessica A. Malloy, Rebecca Raeside, Si Si Jia, Anna C. Singleton, Mariam Mandoh, Allyson R. Todd, Tian Wang, Nicole K. Halim, Karice Hyun, Julie Redfern

**Affiliations:** 1 Westmead Applied Research Centre, Faculty of Medicine and Health, The University of Sydney, Sydney 2145, Australia; rebecca.raeside@sydney.edu.au (R.R.); sisi.jia@sydney.edu.au (S.S.J.); anna.singleton@sydney.edu.au (A.C.S.); mariam.mandoh@sydney.edu.au (M.M.); Allyson.R.Todd@student.uts.edu.au (A.R.T.); twan6100@uni.sydney.edu.au (T.W.); nhal7837@uni.sydney.edu.au (N.K.H.); karice.hyun@sydney.edu.au (K.H.); julie.redfern@sydney.edu.au (J.R.); 2 Prevention Research Collaboration, Charles Perkins Centre, Sydney School of Public Health, The University of Sydney, Sydney 2006, Australia; 3 Menzies Centre for Health Policy, Sydney School of Public Health, Faculty of Medicine and Health, The University of Sydney, Sydney 2006, Australia; alice.gibson@sydney.edu.au; 4 Discipline of Nutrition and Dietetics, Faculty of Medical and Health Sciences, The University of Auckland, Auckland 1011, New Zealand; r.roy@auckland.ac.nz (R.R.); jmal232@aucklanduni.ac.nz (J.A.M.); 5 ANZAC Research Institute, Concord Repatriation General Hospital, The University of Sydney, Sydney 2137, Australia; 6 The George Institute for Global Health, The University of New South Wales, Camperdown 2006, Australia

**Keywords:** food environment, online food delivery, diet, nutrition, take out, fast food, young adult, adolescent

## Abstract

The demand for convenience and the increasing role of digital technology in everyday life has fueled the use of online food delivery services (OFD’s), of which young people are the largest users globally. OFD’s are disrupting traditional food environments, yet research evaluating the public health implications of such services is lacking. We evaluated the characteristics and nutritional quality of popular food outlets on a market-leading platform (UberEATS^®^) in a cross-sectional observational study conducted in two international cities: Sydney (Australia) and Auckland (New Zealand). A systematic search using publicly available population-level data was used to identify geographical areas with above-average concentrations (>30%) of young people (15–34-years). A standardized data extraction protocol was used to identify the ten most popular food outlets within each area. The nutritional quality of food outlets was assessed using the Food Environment Score (FES) (range: −10 ‘unhealthiest’ to 10 ‘healthiest’). Additionally, the most popular menu items from each food outlet were classified as discretionary or core foods/beverages according to the Australian Dietary Guidelines. The majority of popular food outlets were classified as ‘unhealthy’ (FES range −10 to −5; 73.5%, 789/1074) and were predominately takeaway franchise stores (59.6%, 470/789, e.g., McDonald’s^®^). 85.9% of all popular menu items were discretionary (n = 4958/5769). This study highlights the pervasion and accessibility of discretionary foods on OFD’s. This study demonstrated that the most popular food outlets on the market-leading online food delivery service are unhealthy and popular menu items are mostly discretionary foods; facilitating the purchase of foods of poor nutritional quality. Consideration of OFD’s in public health nutrition strategies and policies in critical.

## 1. Introduction

Obesity is among the biggest health challenges facing the world [1], in particular for adolescents and young adults [2]. The major risk factors driving the global burden of obesity are diet-related [3]. Dietary risk factors for obesity and chronic diseases include diets high in discretionary foods (i.e., energy-dense, nutrient-poor foods high in saturated fat, added sugars and sodium) [4]. Food retail environments influence dietary behaviors and obesity prevalence [5]. Evidence for the causal relationship between food environments and obesity is limited [6,7]. However, recent research has demonstrated associations between restaurant and takeaway food consumption with increased discretionary food intake [8]. Takeaway foods are one of the fastest-growing categories in online retail sales index, experiencing a 99% annual growth in Australia [9]. Young people (15-to-34-years-old) in Australia are spending on average AUD$100/week eating at restaurants or ordering takeaway foods [10]. Internet and smartphones in the modern food environment have emerged as tools to enable immediate access to many food outlets to order food directly to a residential home or workplace [11].

Online food delivery services (OFD’s) are defined as websites or smartphone applications that allow customers to order menu items from food outlets for pick-up or delivery by freelance couriers [12]. To access these services, customers create a personal account with credit or debit card details for automatic payment and their delivery address. If a customer selects delivery, they can generally track the progress of their order on the website or app. In 2020, the global revenue from OFD’s is projected to be US$2,082 million [12], with Uber Eats (UberEATS^®^, Uber Technologies Inc) accounting for 30% of the market [12]. Globally, young people (18–34-years) are the main users (48.4%) of OFD’s [13]. In Australia and New Zealand, over 25% of young people (15–34 years) are reported users [14]. A recent Australian study found that OFD’s are commonly used by working young adults who have higher disposable incomes [15]. The recent increase in the popularity of OFD’s is challenging traditional food retail environments by changing the geographical reach and availability of restaurant and takeaway foods [16]. The current COVID-19 (coronavirus disease of 2019, a disease caused by the SARS-CoV2 virus [17]) pandemic and government restrictions related to social distancing may have further accelerated the demand for online food delivery. Subsequently, some restaurants and food outlets have increased their geographical delivery distance to reach more people [18].

The rapidly escalating demands for the convenience of OFD’s have driven research in the consumer service-related fields [19,20,21,22,23], yet there has been a paucity of research evaluating the public health implications of online food delivery services [24]. A recent cross-sectional study examined the prevalence and frequency of using OFD’s in five countries in 2018. This study found that 15% of respondents had used online food delivery in the past seven days. Moreover, it showed that 35% of all meals purchased away-from-home were through OFD’s (40% among Australian responders). Respondents who were male and younger, among other sociodemographic characteristics, had greater odds of using OFD’s [25]. Another cross-sectional study in three countries demonstrated a large variety of food types consumers can purchase using OFD’s [16]. Most food types were considered unhealthy, and there was not a higher proportion of healthy food types available for consumers living in lower socioeconomic neighborhoods [16]. The food types analyzed were based on keywords provided on the food delivery outlet, and the study did not independently assess the nutritional quality of food outlets or menu items. Both of these studies have provided crucial insights into the prevalence and frequency of OFD’s, and both reveal the vast number of food outlets now readily accessible at our fingertips. Important gaps remain about the nutritional quality of food outlets and the food they provide, in order to evaluate the public health implications of online food delivery services.

The primary aim of this present study was to evaluate the healthiness and geographical reach of popular food outlets and the nutritional quality menu items on a market-leading OFD platform, in areas with high concentrations of young consumers (15–34-years) across two high-income cities, Sydney, Australia and Auckland, New Zealand. A secondary aim was to examine the differences between food outlet characteristics and the socioeconomic disadvantage level within each city.

## 2. Materials and Methods

### 2.1. Study Design and Context

A cross-sectional observational study was conducted in two international cities: Sydney, Australia and Auckland, New Zealand. These two cities were purposely selected to compare different populations of high-income countries with similar proportions of young people—the primary users of OFD’s. Sydney and Auckland have populations of approximately 4.8 million and 1.5 million people, respectively [26,27]. In Sydney, 29% of the population in 2016, and in Auckland, 27.3% of the population in 2018 were aged 15–34 years [26,27]. In Sydney, young people, aged 15–34 years, are the main users of online food delivery services, with 29.7% and 28.7% of Millennials (born 1981–1996) and Generation Z (born 1997–2012), in Australia using online food delivery services, respectively [14]. Over the last 12-months, there has been a 13% increase in online food delivery services usage amongst young people in Australia [14]. Similarly, in Auckland, 17.8% of 18–24-year-olds and 29.1% of 25–34 years-old’s are using online food delivery services. This also indicates that young people make up the largest proportion of users in New Zealand [28].

### 2.2. Identification of Online Food Delivery Service

Uber Eats was selected as the OFD choice because it remains the market leader in Australia [14] and New Zealand [28]. Recently, it was estimated that 11.5% of all of the people living in Australia use Uber Eats [14]. As of May 2018, the usage share of Uber Eats amounted to an estimated 30% (highest) of the entire online food delivery segment in both Australian and New Zealand [12,28].

### 2.3. Selection of Geographical Areas

A systematic search using publicly available population-level data was used to identify geographical areas and associated suburbs with above-average concentrations of young people. Suburbs are defined as specific geographical subdivisions of a city, including bother inner and outer regions of the cities. Geographical areas were Local Government Areas (LGAs) [29] and regional councils [26] in Sydney and Auckland, respectively, that met the following inclusion criteria: (i) had Uber Eats delivery coverage [30,31]; and (ii) had above average populations (>30%) of young people (15–34-years) using national Census data [26,27]. In Sydney, 13 LGAs (covering 233 suburbs) met the above criteria, and in Auckland, Auckland Council (covering 186 suburbs) met the above criteria.

### 2.4. Identification of the Most Popular Food Outlets

All suburbs (233 Sydney, 186 Auckland) were searched between 9 to 25 February 2020 for Sydney and 23 June to 31 July 2020 for Auckland. Searches were conducted using the Australian, and New Zealand Uber Eats websites [30,31]. To avoid possible bias introduced by previous usage of the services, researchers were not logged into personal Uber Eats accounts at the time of data extraction. In the ‘delivery address location’ bar, only the suburb was entered by searching the suburb name, state and postcode, e.g., Sydney NSW 2000 or Auckland Central 1010. No specific residential or workplace address was entered. For consistency, the delivery time was set for 6.00 PM–6.30 PM on the day of searching as this is when food outlets operate for dinner. The Uber Eats website allows customers to select ‘delivery now’ or ‘schedule for later’. The ‘schedule for later’ section is divided into 30-min increments. Uber Eats then provides customers with a ‘popular near you’ section, which is a list of the most popular food outlets who are willing to deliver to the customers delivery location. The ‘popular near you’ list is dependent on delivery coverage and the geographical radius set by individual food outlets. Researchers extracted data on the first ten most popular food outlets nearest to each suburb’s delivery address location (SRP, JM, RR, AS, MM, AT, SJ). Duplicate food outlets were identified, and a set of unique food outlets were compiled for analysis. Unique food outlets were defined as having a distinct physical location. For example, different physical locations of franchise stores (takeaway and restaurant/café) were considered as unique. i.e., McDonald’s^®^ Auburn and McDonald’s^®^ Bexley. Data outcomes are described below.

### 2.5. Data Extraction

Using a standardized protocol, the following characteristics of the most popular food outlets were extracted: Uber Eats food outlet categories (e.g., categories such as pizza, fast food, healthy, desserts, Thai, bubble tea), cost of delivery (AUD$ or NZ$), number of customer reviews (if >500 reviews listed as ‘500+’) and ratings (out of 5). The geographical delivery distance was determined using Google Maps to calculate the shortest delivery route (kilometers, km) by road between the food outlet and the delivery suburb [32]. Four authors (SRP, JM, TW, NH) searched the delivery routes between a food outlet address and the delivery suburb using Google Maps. For consistency, no specific residential address was entered, only the delivery suburb (i.e., Sydney NSW 2000). On Google Maps, this results in a location pin dropped in the geographical center of the suburb. When several delivery routes were recommended, the shortest geographical distance was recorded. Any geographical delivery distances greater than 10 km were cross-checked by the lead author (SRP). The most popular menu items were extracted from the ‘most popular’ section on each food outlet’s menu. Commonly, food outlets list five most popular menu items, with some listing up to ten. Table 1 provides a summary and definition of these data features. A summary of outcome measures and definitions are provided in Table 1.

### 2.6. Outcome Measures

The primary outcomes of this study were to evaluate the nutritional quality and geographical reach of popular food outlets and menu items. A secondary aim was to examine the differences between food outlet characteristics and the socioeconomic disadvantage level within each city.

#### 2.6.1. Healthiness of Popular Food Outlets

The healthiness of food outlets was assessed using an adapted version of the Food Environment Score (FES) tool, which assigns classification and a healthiness score to food outlets [33]. The FES is a food outlet classification and healthiness scoring tool developed by expert consensus and agreement for food outlet types in Australian residential communities [33]. The FES uses a classification system and 20-point scoring tool, ranging from −10 (least healthy) to +10 (mostly healthy) [33]. For the current study, the food outlet ‘healthiness’ classifications were based on a recent Australian study, which used an adapted FES classification and health score [34]. Using the adapted FES, food outlets were classified into 18 food outlet types [34], scored and classed by ‘healthiness’ into three groups: healthy (FES range +5 to +10); less healthy (FES range −4 to +4) and unhealthy (FES range −10 to −5). Classification, scoring and grouping were conducted by three university-qualified dietitians (JM, TW, SJ). The lead author, who is also university-qualified dietitian (SRP) conducted a 20% cross-check of the classification and scoring and discrepancies were resolved by consensus.

#### 2.6.2. Nutritional Quality of Most Popular Menu Items at Included Outlets

The most popular menu items were classified as discretionary or a core food based on their description. Discretionary foods are defined in the Australian Dietary Guidelines as “*foods and drinks not necessary to provide the nutrients with the body needs, but that may add variety. However, many of these are high in saturated fats, sugars, salt and/or alcohol, and are therefore described as energy-dense*” [35]. Core foods defined as foods or combination of foods from the five food groups: vegetables and legumes/beans, fruit, grain (cereal) foods, mostly wholegrain and/or high cereal fiber varieties, lean meats and poultry, fish, eggs, tofu, nuts and seeds and legumes/beans and milk, yoghurt cheese and/or alternatives, mostly reduced fat) [35]. The Australian Bureau of Statistics (ABS) discretionary food list was used as a reference [36]. This list was informed by the 2013 Australian Dietary Guidelines, which aligns with the New Zealand Eating and Activity Guidelines [37] and supporting documents [35]. The ABS used additional criteria based on nutrient profiles to identify foods as a discretionary or core food. For the present study, nutrient profiles for most popular menu items were not available. All sugar-sweetened beverages (SSBs) (e.g., soft drinks, energy and sports drinks, bubble teas, e.g., pearl milk tea) and intense sweetened beverages (e.g., diet soft drinks) were classified as discretionary. When the data extracted from Uber Eats was insufficient to determine whether the menu item was discretionary, a conservative approach was used, and the menu item was classified as core food. For example, using the ABS list, mixed noodle dishes such as pad Thai, ramen or stir fry noodles, were considered discretionary if the saturated fat is >5 g/100 g. For the current study, this menu item was classified as a core food as the nutritional contents were unknown. When a popular menu item contained a discretionary component (e.g., katsu (fried) chicken sushi or a ‘meal deal’ with hot chips or SSB), the menu item was classified as discretionary. The discretionary classification was conducted by three university-qualified dietitians (JM, TW, SJ). The lead author, who is also university-qualified dietitian (SRP) conducted a 20% cross-check of the classification and discrepancies were resolved by consensus.

To determine whether the proportion of discretionary foods differed by the FES categories of healthiness (unhealthy, less healthy, healthy), the proportion of discretionary foods was stratified by FES score and also by the Uber Eats category of ‘healthy’.

#### 2.6.3. Socioeconomic Disadvantage Level

In Sydney, the Socioeconomic Indexes for Areas (SEIFA) Index of Relative Socioeconomic Disadvantage (IRSD) was used to identify the quintile of socioeconomic disadvantage of (i) the delivery suburb; and (ii) the suburb in which the food outlet was physically located [38]. SEIFA IRSD provides measures of socioeconomic conditions by geographic area ranks areas in Australia according to relative socioeconomic disadvantage. In Auckland, the 2018 New Zealand Index of Deprivation (NZ Dep) was used, which combines the following dimensions of deprivation: communication, income, employment, qualifications, owned home, support, living space, and living condition [39]. The SEIFA IRSD and NZ Dep allocate each suburb a decile of deprivation. For this study, deciles were aggregated into quintiles, with quintile 1 (decile 1 and 2) representing the 20% least disadvantaged areas and the fifth quintile (decile 9 and 10) represents the 20% most disadvantaged areas in Sydney and Auckland.

### 2.7. Data Analysis

Descriptive statistics were used to evaluate the food outlet characteristics, the healthiness of food outlets and the nutrition quality of their most popular menu items. Normality of the continuous variables were tested using the Kolmogorov-Smirnov test and the data with skewed distribution were summarized as medians and interquartile intervals. Kruskal-Wallis tests were used for continuous variables, and Chi^2^-tests were used for categorical variables to examine differences between food outlet characteristics, healthiness of food outlets and the nutrition quality of their most popular menu items with the socioeconomic disadvantage level within each suburb. Further, post hoc multiple comparisons were performed for the significant differences using the Dunn test [40]. All analyses were undertaken using SAS version 9.4 (SAS Institute Inc, Cary, NC, USA).

## 3. Results

### 3.1. Identification of Most Popular Food Outlets and Menu Items

**Figure 1:**
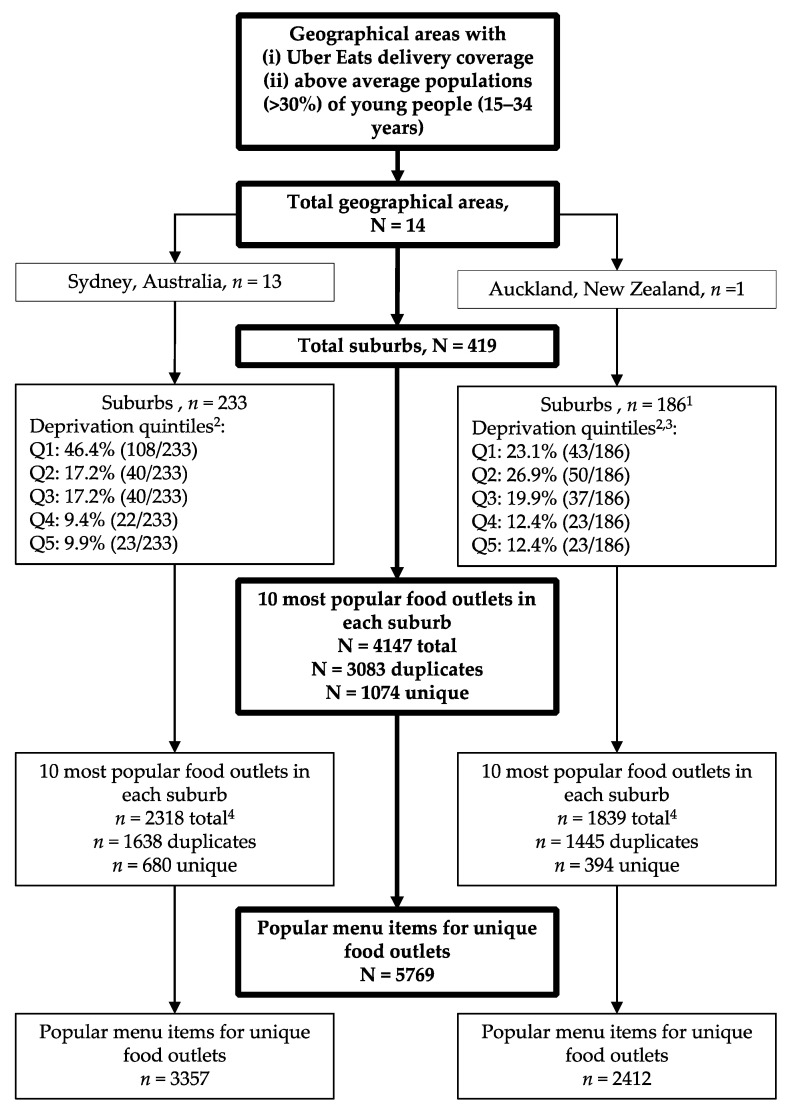
Flow diagram of included unique food outlets and most popular menu items in Sydney, Australia and Auckland, New Zealand. ^1^ Suburbs with the Uber Eats Auckland service area; ^2^ Q1 least disadvantaged suburbs and Q5 most disadvantaged suburbs; ^3^ Ten Auckland suburbs did not have a dep index available (10/186, 5.4%);^4^ Nine suburbs had less than ten most popular food outlets.

Based on areas with >30% population of young people (15–34 years) and Uber Eats delivery coverage, there were 13 LGAs in Sydney, Australia and one regional council in Auckland, New Zealand, which contained 233 suburbs and 186 suburbs, respectively, (total 419; Figure 1). The total population of Sydney’s 13 LGAs was approximately 1.6 million people, and the geographical area covered approximately 420 km^2^ [27]. Uber Eats did not have full delivery coverage across Auckland regional council, which has a population of 1.6 million people and covers a geographical area of approximately 4940 km^2^ [26]. The 186 suburbs covered five districts in the Auckland metropolitan area. In Sydney and Auckland, the majority of included suburbs were classified as least disadvantaged (Q1 and Q2), with 64.1% (148/233) in Sydney and 50% (93/186) in Auckland.

After extracting data from all 419 suburbs regarding the ten most popular food outlets, there were 4157 food outlets (2318 in Sydney and 1839 in Auckland). A food outlet could be among the most popular in multiple suburbs, resulting in many duplicate food outlets. Therefore, accounting for duplicates, there were 1074 unique food outlets (680 in Sydney and 394 in Auckland) with 5769 most popular menu items (3357 in Sydney and 2412 in Auckland).

### 3.2. Most Popular Food Outlet Characteristics and Geographical Reach

The characteristics of the unique food outlets available on Uber Eats in Sydney, Australia and Auckland, New Zealand are presented in Table 2. The median food outlet rating and reviews were comparable between the two cities. Food outlets delivered to more suburbs in Auckland compared to Sydney (3 vs. 2 suburbs, respectively). The median delivery costs were similar between the two cities; however, the range of the delivery cost was greater in Sydney due to a free delivery promotion at Domino’s Pizza (takeaway franchise store). The median delivery distance was 3.00 km and 3.20 km in Sydney and Auckland, respectively. Only 11.9% and 10.3% of the unique delivery routes were ≤1 km in Sydney and Auckland, respectively. Most delivery routes were between 1.1–5.0 km (80.0%, in Sydney vs. 73.9% in Auckland). There were more delivery routes between 5.1–10.0 km in Auckland (15.4%) compared to Sydney (7.6%). Less than 1% of delivery routes were >10 km in both Sydney and Auckland (0.4% in Sydney and Auckland).

### 3.3. Nutritional Quality of Food Outlets

#### 3.3.1. Healthiness of Popular Food Outlets

In both cities, most food outlets were scored as unhealthy using the FES tool (73.4% in Sydney and 73.6% in Auckland) (Table 2). A further 21.5% (146/680) and 22.1% (87/394) were scored as less healthy in Sydney vs. Auckland, respectively. The remaining food outlets were scored healthy (5.1%, 35/680 in Sydney vs. 4.3%, 17/394 in Auckland).

In both cities, the most popular food outlet classification was ‘takeaway franchise stores.’ There were more takeaway franchise stores in Auckland (54.3%) compared to Sydney (37.7%) (Table 2). In Sydney, the 257 takeaway franchise stores were from 20 fast-food companies. The most common takeaway franchise store was McDonald’s^®^ (54/680, 8.4%), followed by Subway (52/680, 7.6%), Oporto (42/680, 6.2%) and Dominos (19/680, 2.8%). In Auckland, the majority of the 214 franchise stores were from 11 fast-food companies. The most common takeaway franchise store was Subway (46/394, 11.7%), followed by McDonald’s^®^ (40/394, 10.2%), Burger King (24/394, 6.1%) and Hell Pizza (20/394, 5.1%). In Sydney, the next most common food outlet classification was takeaway local independent food outlets (29.7%), followed by restaurant/café local independent food outlets (20.7%). In Auckland, restaurant/café local independents accounted for 15.2% of food outlets, followed by takeaway local independent food outlets (14.5%). Salad/sushi food outlets accounted for less than 5% of all food outlets in Sydney (4.7%) and Auckland (3.8%).

#### 3.3.2. Nutritional Quality of Most Popular Menu Items at Included Outlets

The majority of the most popular menu items were classified as discretionary (84.3%, 2839/3357 in Sydney vs. 88.2% 2128/2412 in Auckland) (Table 2). In Sydney, SSB accounted for 15.9% (532/3357) of the most popular menu items. In contrast, in Auckland, SSBs accounted for only 1.0% (22/2412) of the most popular menu items. However, in Auckland, 29.0% (700/2128) of most popular menu items were meal deals, which included either hot chips or SSB. Whereas in Sydney, meal deals which included either hot chips or SSB accounted for 8.6% (289/3357) of the most popular menu items. Core foods accounted for 15.7% (527/3357) and 11.8% (284/2412) of popular menu items in Sydney and Auckland, respectively.

Table 3 compares the proportion of discretionary foods in the most popular menu items by the healthiness of the food outlet using the FES and also by the Uber Eats category of ‘healthy’. This demonstrates, that in both Sydney and Auckland, food outlets that were classified as unhealthy (FES range −10 to −5) had mostly discretionary foods as popular menu items (95.7% in Sydney and 81.2% in Auckland). Food outlets that were classified as less healthy (FES range −4 to 4) and healthy (FES range 5 to 10) had increasing proportions of core foods as popular menu items in both cities. It also highlights the discrepancies between food outlets categorized as ‘healthy’ on Uber Eats vs. FES classification of healthiness. In Sydney, a total of 110 from 680 food outlets were categorized as ‘healthy’ on Uber Eats (Table 3). However, using the FES, 96 out of the 110 food outlets were classified as unhealthy (FES range −10 to −5) and 469/532 (88.1%) of their most popular menu items were classified as discretionary foods. In Auckland, a total, 66/394 food outlets, classified themselves as ‘healthy’ on Uber Eats. However, 154 (96 in Sydney and 57 in Auckland) food outlets were classified as unhealthy according to the FES (range −10 to −5). Of the 330 most popular menu items from the 66 restaurants that were categorized as ‘healthy’ on Uber Eats, 243 (73.6%) were classified as discretionary. In Sydney, food outlets that were classified as ‘healthy’ on Uber Eats that were classified as healthy (FES range 5 to 10), had higher proportions of core foods as popular menu items. However, in Auckland, popular menu items had high proportions of discretionary foods across all FES categories.

### 3.4. Differences Between Physical Locations of Food Outlet by Deprivation Quintiles and Food Outlet Characteristics

Differences between deprivation quintiles and food outlet characteristics are presented in Table 4. In Sydney, 60% (407/680) of the food outlets were located in the least disadvantaged suburbs (Q1 and Q2). In contrast, food outlets were more evenly distributed across deprivation quintiles in Auckland. The deprivation quintiles of the physical food outlet location were similar to the deprivation quintiles of the delivery suburb in Sydney (*p* < 0.0001) and Auckland (*p* < 0.0001). This indicates that food outlets are delivering within the suburb they are located or to similar surrounding suburbs. There were differences between delivery costs, delivery distance and deprivation quintiles of the physical food outlet location in Sydney and Auckland. Slight increases in delivery costs (Sydney: *p* < 0.0001; Auckland: *p* < 0.0001) and delivery distance (Sydney: *p* < 0.0001; Auckland: *p* = 0.0004) were observed with increasing quintiles of deprivation. In Sydney, post hoc comparisons demonstrated significant differences in delivery costs (Q1 versus Q2; Q1 versus Q3; Q1 versus Q4; and Q1 versus Q5) and delivery distances (Q1 versus Q2; and Q2 versus Q3) between deprivation quintiles. In Auckland, there were significant differences in delivery costs (Q1 versus Q4; Q2 versus Q4; Q3 versus Q4; and Q4 versus Q5) and delivery distances (Q1 versus Q4; Q3 versus Q4; and Q4 versus Q5) between deprivation quintiles. In Auckland, there were differences between the healthiness of food outlets (FES score) and the deprivation quintiles of the physical food outlet location (*p* = 0.0277). Post hoc comparisons demonstrated food outlets with healthier FES scores were located significantly more in the least disadvantaged suburbs (Q1) compared to the most disadvantaged suburbs (Q5). There were no differences in the proportion of discretionary menu items across the physical location deprivation quintiles in both Sydney and Auckland. 

## 4. Discussion

This study is the first to evaluate the characteristics and healthiness of the most popular food outlets in addition to the nutritional quality of their most popular menu items on a market-leading online food delivery platform. Overall, we found that almost three-quarters of the most popular food outlets were classified as unhealthy using the FES, with half of the food outlets in Auckland and a third of food outlets in Sydney being classified as takeaway food franchise stores such as McDonald’s^®^ and Burger King^®^. We also found that almost 9 out of 10 of the most popular menu items in both cities were discretionary foods. In Sydney, 1 in 6 of the most popular items were SSBs, and in Auckland, almost 1 in 3 were meal deals which included hot chips or SSBs. Although this study did not directly measure consumption, our investigation of the most popular food outlets, as well as the most popular menu items of these outlets, suggest that a large proportion of users of OFD’s are using it to access and purchase ‘junk’ foods.

An important finding of this study is that the majority (~90%) of delivery distances were greater than 1 km, a distance which has been typically used to define a neighborhood food environment [41,42]. This demonstrates that OFD’s are disrupting traditional food environments by increasing the reach and accessibility of food outlets. Moreover, using OFD’s may further promote sedentary lifestyle behaviors [24]. The impacts OFD’s have on population dietary intake remains an outstanding and crucial question. It is not known whether the use of OFD’s is simply changing the *mode* of purchasing food away from home, i.e., from face-to-face to online, or whether it is changing *what* or *how often* food is purchased away from home. Regardless, our findings combined with the growing demand for OFD’s demonstrate the need to incorporate ‘digital food environments’ into public health nutrition strategies and policies, which to date are still focused on the built environment [43,44].

Unhealthy food outlets and discretionary menu items dominated the sample of food outlets and menu items in this study. This finding aligns with reports released from other OFD’s. For example, a 2019 report from the most frequently used app for OFD’s in the United States of America, ‘Door Dash’^®^ revealed American consumers’ top ordered foods were discretionary foods, including, cheeseburger and fries, pizza, nachos, cheesecake, baby back pork rib, chicken and waffle sliders [45]. Moreover, in the United Kingdom, the leading app, Grubhub^®^, reported that in 2018 over 70% of its customers utilized the app to order quick service or fast-casual foods [46]. As such, it is becoming evident that these energy-dense nutrient-poor discretionary options are the most popular selections to be delivered. However, in contrast to our findings, a report released from Uber Eats Australia stated that ‘*one in five Australians said that they tend to eat healthier when they order in compared to when they cook*.’ Applying the FES to food outlets suggested that those food outlets that were categorized as ‘healthy’ on Uber Eats were mostly ‘unhealthy.’ As such, health claims by food outlets on Uber Eats may be misleading consumers. Further independent research is needed to understand the impact of such services on exposure to unhealthy foods and dietary intake, given OFD’s have strong commercial interests with large fast-food franchises.

Emerging research from other countries have also provided insights into the impacts of online food delivery exposure to unhealthy foods, dietary intake and risk factors for chronic disease. For example, a survey in Xi Hu District, Hangzhou, China found that 42% of the total food outlets in the district provided food delivery services. Of all the food outlets, fast-food restaurants comprised 66% of these providers, potentially increasing the likelihood of exposure to unhealthy food choices [47]. Moreover, a study of 1220 university students in Beijing, China found that a high frequency of online delivery food consumption was associated with a preference for discretionary foods (high fat and high sugar foods), physical inactivity and high body mass index [48]. Consumption was not measured in the current study to draw comparisons. Despite this, geographical areas were selected with high concentrations of young people as they are the predominate users of online food delivery. With a majority of food on these platforms classified as discretionary, our data indicates that young people have increased accessibility to these unhealthy choices, thereby increasing their risk for excess weight gain and obesity [4]. Although, whether there is a casual relationship between neighborhood accessibility to fast-food outlets and obesity remains a topic for debate [49]. OFD’s are furthermore, disrupting the traditional neighborhood food environment and studies have yet to take this into consideration. As OFD’s continue to change accessibility to fast-food and potential consumer behaviors, further research must assess whether there is an association with risk factors for chronic diseases, like obesity amongst young consumers.

A strength of this study is the independent evaluation of the healthiness of food outlets on Uber Eats using the FES. On Uber Eats food outlets were classified as ‘healthy’. Poelman et al. evaluated meal delivery options in three international cities using the pre-defined keywords set by the food outlets [16]. Limited evidence was found for socioeconomic differences of ‘healthy’ options within the three cities. However, this study did not independently assess the healthiness of food outlets. Our evaluation demonstrated most food outlets that were classified as ‘healthy’ on Uber Eats were unhealthy according to the FES. This unhealthy score was further supported by our classification of popular menu items as mostly discretionary from these food outlets. Despite this, we did not evaluate the nutritional quality of the full menu and we used a conservative approach to the classification of discretionary based on limited information. For instance, in the absence of nutrition information, a menu item was counted as core food. As such, popular dishes such as Pad Thai were counted as core food, but, are likely to be discretionary as they are commonly high in energy (856 kJ/100 g), and sodium (527 mg/100 g) [50]. Therefore, it is likely that we have overestimated the proportion of core foods.

There are further limitations to this study that should be acknowledged. We searched and extracted publicly available data from Uber Eats, the market-leading company and most popular online food delivery platform for young people in Australia and New Zealand. However, there are other online food delivery platforms with large usage (e.g., in Australia, Menulog^®^ and Deliveroo^®^ and in New Zealand, Just Eat^®^). Thus, we may have excluded key food outlets as some may only be affiliated with one online food delivery platforms. Furthermore, the Sydney and Auckland data were not collected over the same period and this may affect comparison analysis. We extracted data in Sydney and Auckland during periods of no government restrictions due to the COVID-19 pandemic (excluding international travel and border restrictions). The Sydney data was collected between 9 February to 25 February 2020 before any stay at home, personal movement or gathering restrictions. The Auckland data was collected between 23 June until 31 July 2020. During this time, the New Zealand government was enforcing COVID-19 Alert Level 1, which enforced no restrictions on personal movement or on gatherings. Nonetheless, the COVID-19 pandemic has possibly influenced people’s dietary intake; however, data is not available. Additionally, the geographical delivery distance was calculated based on road distance by car which may have been a longer route than by bicycle. The definition of the most popular food outlets and most popular menu items were assumed to mean the most popular based on customer purchasing and ratings. Evaluating the most popular food outlets may not be representative of all food outlets on Uber Eats; however, this data potentially gives a better indication of what consumers purchase and consume through the online delivery service. The proprietary Uber Eats algorithms for most popular food outlets and menu items are not made publicly available.

## 5. Conclusions

The consumer demand for online food delivery services is rapidly growing. The results from this study suggest there is a digital disruption occurring within traditional built food environments. This study demonstrated that the most popular food outlets on the market-leading online food delivery service are unhealthy and popular menu items are mostly discretionary foods (i.e., ‘junk foods’). While the majority of food outlets in both cities were takeaway franchise stores, a significant proportion of food outlets were local independent takeaway stores. Local independent stores are often exempt from public health nutrition strategies applied to takeaway franchise stores. The proportion of local independent stores found in the current study warrants further investigation into the nutritional quality of menu items. Online food delivery services are proliferating by the accommodating consumer demands for simple, convenient and ‘on-trend’ services, however, appear to facilitate the purchase of foods of poor nutritional quality and requires serious and immediate consideration in public health nutrition strategies and policies.

## Figures and Tables

**Table 1:** Summary and definition of data extracted from food outlets and derivation.

Data Features	Definition
Popular food outlets	Food outlets listed in the ‘popular near you’ section of the Uber Eats website after entering a delivery location (i.e., suburb)
Unique food outlets	A food outlet with a distinct physical location
Rating (/5)	Rating is calculated based on the average ratings a food outlet received for their last 500 rated orders, or all orders they have completed if they haven’t done 500 yet
Reviews	Number of reviews a food outlet received up to 500. If a food outlet has >500 reviews listed as ‘500+’
Number of delivery suburbs	Number of suburbs with delivery coverage for a unique food outlet
Delivery cost ($)	Cost of delivery for an order from a food outlet to a delivery suburb
Delivery distance (km)	Shortest distance between the food outlet and the delivery suburb determined using Google Maps [32]
Unique delivery routes	Unique food outlet multiplied by number of delivery suburbs
Most popular menu items	Menu items that are listed in the first section of a food outlets full menu under the heading ‘most popular’

**Table 2:** Characteristics of food outlets, the healthiness of food outlets and nutritional qualities of most popular menu items available on Uber Eats in Sydney, Australia and Auckland, New Zealand.

Characteristics	Sydney, Australia	Auckland, New Zealand
Number of unique food outlets (n)		
Total number of unique food outlets	680	394
Food outlet ratings, median (IQR)		
Rating (/5)	4.4 (4.3–4.6)	4.5 (4.3–4.6)
Reviews ^1^	323 (170–500+)	245 (122–431)
Delivery details, median (IQR)		
Number of delivery suburbs	2 (1–4)	3 (1–6)
Delivery cost ($AUD/$NZD)	$5.99 ($3.99–$6.99)	$7.99 ($5.99–$7.99)
Delivery distance (km)	3.00 (1.90–4.00)	3.20 (2.00–4.40)
Unique delivery routes ^2^	2318	1839
Unique delivery route >1 km, n (%)	2042 (88.1)	1648 (89.7)
Food outlet classification [FES], n (%)		
Bakery (0)	1 (0.1)	0 (0)
Restaurant/café franchise (0)	4 (0.6)	27 (6.9)
Restaurant/café local independent (0)	141 (20.7)	60 (15.2)
Salad/sushi bar (5)	32 (4.7)	15 (3.8)
Sandwich shop (5)	2 (0.3)	2 (0.5)
Major supermarket (5)	1 (0.1)	0 (0)
Specialty food store—extra foods (−8)	41 (6.0)	19 (4.8)
Take-away local independent (−8)	202 (29.7)	57 (14.5)
Take-away franchise store (−10)	256 (37.6)	214 (54.3)
Food outlets grouped by healthiness, n (%)		
Healthy (FES range 5 to 10)	35 (5.1)	17 (4.3)
Less Healthy (FES range −4 to 4)	146 (21.5)	87 (22.1)
Unhealthy (FES range −10 to −5)	499 (73.4)	290 (73.6)
Most popular menu items, n (%)		
Total number of most popular menu items	3357	2412
Discretionary foods	2830 (84.3)	2128 (88.2)
Core foods	527 (15.7)	284 (11.8)
Deprivation quintiles of physical food outlet location, n (%)	SEIFA IRSD 2016	NZDep 2018 ^3^
Q1 Least disadvantaged suburbs	271 (39.9)	48 (12.2)
Q2	136 (20.0)	109 (27.7)
Q3	113 (16.6)	71 (18.0)
Q4	71 (10.4)	95 (24.1)
Q5 Most disadvantaged suburbs	89 (13.1)	49 (12.4)

n, number; AUD, Australian Dollar; FES, Food Environment Score; IQR, Inter Quartile range; NZD, New Zealand Dollar; km, kilometer; Q, quintile; SEIFA IRSD, Socio-Economic Indexes for Areas Index of Relative Socioeconomic Disadvantage; NZDep, New Zealand Index of Deprivation. ^1^ Food outlets with ratings above 500 were listed as 500+. For the purpose of this study counted as 500; ^2^ Unique delivery route = Unique food outlets × number of delivery suburbs; ^3^ Three suburbs had no NZDep 2018 available; therefore, 22 unique food outlets had missing data (5.6% of total unique food outlet locations for Auckland, New Zealand).

**Table 3:** Proportion of discretionary foods in the most popular menu items by the healthiness of the food outlet using the FES and also by the Uber Eats category of ‘healthy’.

	Food Outlets Grouped by ‘Healthiness’		
	Unhealthy (FES Range −10 to −5)	Less Healthy (FES Range −4 to 4)	Healthy (FES Range 5 to 10)
**Sydney, Australia**			
Most popular menu items, n	2463	723	171
Discretionary foods, n (%)	2358 (95.7)	415 (57.4)	57 (33.3)
Core foods, n (%)	105 (4.3)	308 (42.6)	114 (66.7)
Food outlets categorized as ‘healthy’ on Uber Eats, n	96	5	9
Discretionary foods, n (%)	449 (97.2)	12 (48.0)	8 (17.8)
Core foods, n (%)	13 (2.8)	13 (52.0)	37 (82.2)
**Auckland, New Zealand**			
Most popular menu items, n			
Discretionary foods	1727 (81.2)	358 (16.8)	43 (2.0)
Core foods, n (%)	159 (56.0)	87 (30.6)	38 (13.4)
Food outlets categorized as ‘healthy’ on Uber Eats, n	57	7	2
Discretionary foods, n (%)	212 (74.4)	25 (71.4)	6 (60.0)
Core foods, n (%)	73 (25.6)	10 (28.6)	4 (40.0)

FES, Food Environment Score.

**Table 4:** Differences between the deprivation quintiles of the physical food outlet location and food outlet characteristics available on Uber Eats in Sydney, Australia and Auckland, New Zealand.

	Deprivation Quintiles										
	Least Disadvantaged						Most Disadvantaged				*p*-Value_diff_
	Q1		Q2		Q3		Q4		Q5		
Sydney, Australia, n (%)	271	(39.9)	136	(20.0)	113	(16.6)	71	(10.4)	89	(13.1)	
**Delivery details**											
SEIFA IRSD deprivation quintile of delivery suburb, median (IQR)	1	(1–2)	2	(1–3)	3	(2–3)	3	(1–3)	4	(3–5)	<0.0001
Delivery cost ($AUD), median (IQR)	4.99	(3.99–6.99)	5.99	(3.99–6.99)	5.99	(4.99–7.99)	5.99	(4.99–7.99)	5.99	(4.99–7.99)	<0.0001
Delivery distance (km), median (IQR)	2.90	(1.7–3.8)	2.90	(1.9–4.0)	3.20	(2.3–4.2)	3.00	(2.0–4.3)	3.20	(1.8–4.1)	<0.0001
**Food outlet ‘healthiness’ score**											
‘Healthiness’ score, median (IQR)	−8	(−10–0)	−8	(−10–0)	−8	(−10–−8)	−8	(−10–0)	−8	(−10–−8)	0.2307
Unhealthy (score < −4), n (%)	185	(68)	100	(75)	88	(78)	52	(72)	74	(84)	0.1307
Less healthy (score −4 to 4), n (%)	67	(25)	29	(21)	20	(18)	17	(24)	13	(15)	
Healthy (score > 4), n (%)	20	(7)	6	(4)	5	(4)	3	(4)	1	(1)	
**Most popular menu items**											
Proportion (%) of discretionary menu items, median (IQR)	100	(66.7–100)	100	(80–100)	100	(80–100)	100	(80–100)	100	(80–100)	0.6167
**Auckland, New Zealand, n (%)**	48	(12.2)	109	(27.2)	71	(18.0)	95	(24.1)	49	(12.4)	
**Delivery details**											
NZDep2018 deprivation quintile of delivery suburb ^1^, median (IQR)	1	(1–2)	2	(1–2)	3	(2–3)	3	(2–4)	4	(3– 5)	<0.0001
Delivery cost ($NZD), median (IQR)	7.99	(4.99–7.99)	7.99	(5.99–7.99)	6.99	(5.99–7.99)	7.99	(6.99–7.99)	7.99	(5.99–7.99)	<0.0001
Delivery distance (km), median (IQR)	3.00	(1.8–4.2)	3.20	(2.0–4.5)	2.80	(1.9–4.4)	3.60	(2.4–4.7)	2.8	(2.1–4.1)	0.0004
**Food outlet ‘healthiness’ score**											
‘Healthiness’ score, median (IQR)	−8	(−10–0)	−10	(−10–0)	−10	(−10–−8)	−10	(−10–−8)	−10	(−10–−8)	0.0277
Unhealthy (score < −4), n (%)	27	(56)	75	(69)	55	(77)	75	(79)	42	(86)	0.0537
Less healthy (score −4 to 4), n (%)	18	(38)	29	(27)	14	(20)	16	(17)	5	(10)	
Healthy (score > 4), n (%)	3	(6)	5	(5)	2	(3)	4	(4)	2	(4)	
**Most popular menu items**											
Proportion (%) of discretionary menu items, median (IQR)	95.0	(80–100)	85.7	(70–100)	100	(80–100)	100	(80–100)	100	(85.7–100)	0.0748

Q, quintile; n, number; IQR, interquartile range; AUD, Australian Dollar; NZD, New Zealand Dollar; km, kilometer; SEIFA IRSD, Socio-Economic Indexes for Areas Index of Relative Socioeconomic Disadvantage; NZDep, New Zealand Index of Deprivation. ^1^ Three suburbs had no NZDep 2018 available, therefore, 22 unique food outlets had missing data for deprivation quintile of physical food outlet location (5.6% of total unique food outlet locations for Auckland, New Zealand) and ten delivery suburbs had no NZDep 2018 available, therefore, 100 unique delivery routes had missing data for deprivation quintile of delivery suburb (5.4% of total unique delivery routes for Auckland, New Zealand).
